# A new horizon of moyamoya disease and associated health risks explored through RNF213

**DOI:** 10.1007/s12199-015-0498-7

**Published:** 2015-12-10

**Authors:** Akio Koizumi, Hatasu Kobayashi, Toshiaki Hitomi, Kouji H. Harada, Toshiyuki Habu, Shohab Youssefian

**Affiliations:** Department of Health and Environmental Sciences, Graduate School of Medicine, Kyoto University, Yoshida Konoe-cho, Sakyo-ku, Kyoto, 606-8501 Japan; Department of Preventive Medicine, St. Marianna University School of Medicine, Sugao, Miyamae-ku, Kawasaki, 216-8511 Japan; Laboratory of Nutritional Sciences, Department of Food Science and Nutrition, Mukogawa Women’s University, Ikebirakicho 4-46, Nishinomiya, 663-8558 Japan; Laboratory of Molecular Biosciences, Graduate School of Medicine, Kyoto University, Yoshida Konoe-cho, Sakyo-ku, Kyoto, 606-8501 Japan

**Keywords:** Moyamoya disease, *RNF213* R4810K, Asian founder mutation, Angiogenesis, Hypoxia

## Abstract

The cerebrovascular disorder moyamoya disease (MMD) was first described in 1957 in Japan, and is typically considered to be an Asian-specific disease. However, it is globally recognized as one of the major causes of childhood stroke. Although several monogenic diseases are known to be complicated by Moyamoya angiopathy, the ring finger protein 213 gene (*RNF213*) was identified as a susceptibility gene for MMD. *RNF213* is unusual, because (1) it induces MMD with no other recognizable phenotypes, (2) the *RNF213* p.R4810K variant is an Asian founder mutation common to Japanese, Korean and Chinese with carrier rates of 0.5–2 % of the general population but a low penetrance, and (3) it encodes a relatively largest proteins with a dual AAA+ ATPase and E3 Ligase activities. In this review, we focus on the genetics and genetic epidemiology of *RNF213*, the pathology of *RNF213* R4810K, and the molecular functions of RNF213, and also address the public health contributions to current unresolved issues of MMD. We also emphasize the importance of a more updated definition for MMD, of qualified cohort studies based on genetic epidemiology and an awareness of the ethical issues associated with genetic testing of carriers.

**Electronic supplementary material:**

The online version of this article (doi:10.1007/s12199-015-0498-7) contains supplementary material, which is available to authorized users.

## Introduction

**Fig. 1:**
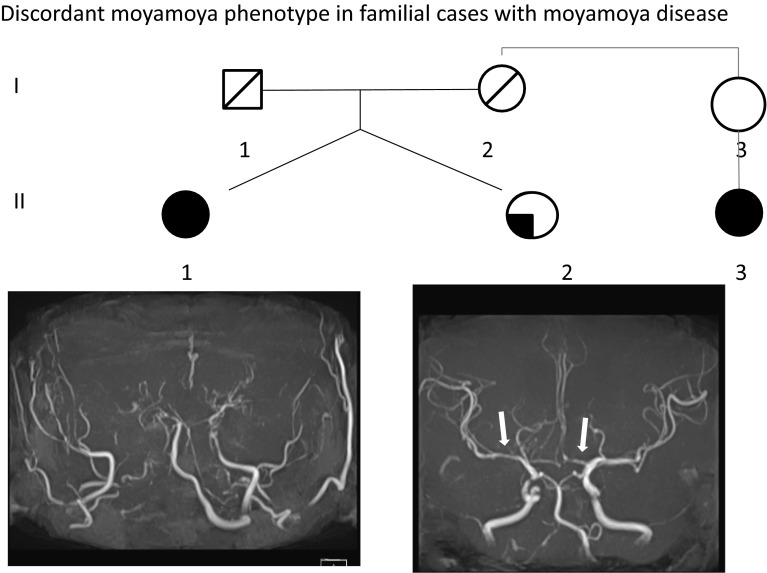
Moyamoya angiopathy. An identical twin first-born twin sister (*II-1*), who developed MMD at the age of 36 years, had stenosis in her anterior and middle cerebral arteries bilaterally and underwent surgery. The second-born twin sister (*II-2*) is represented with a solid quadrant. Magnetic resonance angiography (*lower panels*) was taken when they were 55 years old. Patient *II-2* showed stenosis (*arrows*). Their mother (*I-2*) died when she was 71 years old from cerebral infarction. Her niece also developed MMD (*II-3*). Subjects *I-3*, *II-1*, *II-2*, and *II-3* all shared the WT/R4810K genotype. We assumed the carrier status for *I-2*, *I-3*, and *II-2* in our linkage analysis. Due to the rarity of the disease gene, we assumed that *I-2* is a carrier of the MMD-associated gene. This pedigree is simplified from the original pedigree 14 [12]

Moyamoya disease (MMD) is a steno-occlusive disease of the cerebral arteries, involving smooth muscle cell proliferation with intima hyperplasia causing arterial stenosis and occlusion around the circle of Willis [1, 2] (Fig. 1). This, in turn, stimulates the compensatory development of collateral vessels, which have a “Puff of Smoke” (Moyamoya in Japanese) appearance in cerebral angiography [3].

MMD is currently recognized as one of the major causes of stroke in children worldwide [4, 5]. Natural disease progression leads to cerebral hemorrhage or cerebral infarction, so early diagnosis and intervention before the establishment of a neurological deficit are essential for improved social adaptation of pediatric patients [6]. Nationwide epidemiological surveys are available in Japan and Korea because of the existence of registration programs. The prevalence and annual incidence of MMD in Japan were reported to be 10.5 and 0.94 per 100,000, respectively, while in Korea these figures were 18.1 and 4.3 per 100,000, respectively, in 2013 [7]. An estimated 100–15 % of MMD patients have family histories [8].

Several monogenic genetic diseases are known to lead to the development of MMD as a complication, referred to as moyamoya syndrome (Table 1). In such diseases, MMD is not the major phenotypic presentation, but it appears to develop in some but not all cases with low penetrance. A comprehensive review of the genetics of MMD associated with monogenic gene diseases has recently been published [9]. Impaired biological processes (signal transduction, chromatin remodeling, DNA repair, inflammation, hemostasis, and vascular smooth muscle cell coagulation), attributable to mutations of associated genes, have given insights into the mechanisms by which the mutations elevate the risk of MMD. However, no consolidated pathological process for MMD development has yet been proposed.

**Table 1:** Single gene diseases showing co-morbidity with moyamoya angiopathy

Biological processes	Molecular pathology	Disease	References		Gene
Signal transduction	Ras signal pathway	Type I neurofibromatosis	[98–101]	*NF1*	[117, 118]
		Noonan syndrome	[102, 103]	*BRAF*	[119]
				*KRAS*	
				*PTPNII*	
				*RAFI*	
				*SOSII*	
		Costello syndrome	[104, 105]	*HRAS*	[120]
	Notch signal pathway	Alagille syndrome	[106, 107]	*JAG1*	[72]
				*NOTCH2*	
	Wnt signal pathway	Robinow syndrome	[108, 109]	*ROR2*	[121]
Chromatin remodeling Cell cycle, DNA repair	Cell cycle	Schimke immuno-osteo dysplasia	[110, 111]	*SMARCAL1*	[122]
		MOPDII	[112, 113]	*PCNT*	[112]
		Seckel syndrome	[114]	*ATR*	[123]
				*RBBP8*	
				*CENPJ*	
				*CEP63*	
				*NIN*	
DNA repair Angiogenesis	BRCA1 complex BRISC complex	X-linked moyamoya syndrome	[88]	*BRCC3*	ibid. [88]
Inflammation	Inflammation activated thrombosis	Sneddon’s syndrome	[66–69]	*CECR1*	[124]
	Excessive Type I interferon production	Aicardi–Goutieres syndrome	[65]	*SAMHD1*	[65]
				*TRX1*	
				*ACP5*	
Vascular smooth muscle cell dysfunction	eNOS production	Moyamoya and achalasia syndrome	[71]	*GUCY1A3*	ibid. [71]
	Excess proliferation	Thoracic aortic aneurysm and dissection	[70]	*ACTA2*	ibid. [70]
Coagulopathy	Thrombosis	Sickel cell disease	[73]	*β*-*globin gene*	
		Protein S	[115, 116]	*Protein S*	
		Protein C	[74, 75]	*Protein C*	
		Thrombotic Thrombocytopeic Purpura	[76]	*ADAMTS13*	

The ring finger protein 213 gene (*RNF213*), (mysterin), was recently identified as a susceptibility gene for MMD. *RNF213* is unusual among susceptibility genes, because it induces MMD with no other phenotypic traits. The *RNF213* variant p.R4810K (c.14429G > A, rs112735431, ss179362673, R4810K hereafter) was first reported by the Kyoto group with a high level of association (odds ratio 63.9 95 %, confidence interval 33.9–120.4) [10] and shown to be associated with MMD at large scales [11, 12]. Both R4859K [11] and R4810K [12] correspond to rs112735431, but while R4859K is based on the computer-predicted open-reading frame in the database [11], R4810K is based on the experimental open-reading frame, which was proven by cDNA cloning [12]. Thus, in this review, we use R4810K. Liu et al. [12] later reported that *RNF213* R4810K is a founder variant in East Asian (Japanese, Korean, and Chinese) patients. Indeed, in Japan and Korea, the majority (~80 %) of MMD patients carry at least one allele of *RNF213* R4810K [12–17]. A much larger proportion of carriers with *RNF213* R4810K is known to develop MMD than that of wild-type (WT) subjects, even though most carriers are unaffected by the disease. This can be explained by the effect of environmental or other genetic factors that elevate the risk of MMD in concert with genetic predisposition. Because the total number of these carriers is estimated to be 15 million in Asian countries, the social impact as a single health issue is extremely significant [18].

RNF213 is composed of 5207 amino acids and has an estimated molecular size of 591 kDa. Its large size initially hampered full-length cDNA cloning, which was first achieved in 2011 [12]. Since then, the biochemical and functional characterization of RNF213 has progressed [12, 19–21], especially through the use of mouse gene ablation technology [20, 22, 23], transgenic mouse models [21], and an induced pluripotent stem cell (iPSC) model established from patients with MMD [24].

This review addresses recent research progress in MMD with regard to effective prevention and intervention programs, enabling public health researchers to identify clear public health goals. In particular, it focuses on *RNF213* in terms of the public health aspect of MMD.

## Multiple genetic loci on 17q25.3 in Japanese patients with familial MMD

MMD has two phenotypic characteristics. The first is apparent from the pathological investigation of cerebral arteries, and involves smooth muscle cell proliferation and neointimal formation with thrombi at the occlusive lesion [25, 26]. This characteristic forms the basis for the alternative name of MMD; sontaneous occlusion of the circle of Willis [27, 28]. Confirmation of this characteristic requires tissue samples for pathological examination, and so it is not practical. The second characteristic is the appearance of moyamoya vessels [3] in angiography, which has been widely used as the diagnostic criterion because of the ease of access in a clinical setting [29]. Current diagnostic criteria of MMD require bilateral stenosis and moyamoya vessels to be observed, while cases with stenosis around the circle of Willis, but the absence of moyamoya vessels, or unilateral stenosis are excluded. However, MMD disease progression starts with stenotic lesions, then leads to unilateral MMD, and culminates in bilateral stenosis with the development of collateral vessels [12, 30]. Therefore, these criteria only cover advanced stage MMD, and exclude cases at an earlier disease stage.

To date, five loci have been reported in Japanese MMD cases: 3p24–p26 [31], 6q25 [32], 8q23 [33], and 17q25/17q25.3 [34, 35]. Linkage analyses were applied to all loci, with the exception of 6q25, in which the association of HLA with MMD was conducted [32]. Loci variation is noteworthy because it argues against the epidemiological observation of a single major locus (17q25.3), and because it is linked with the default application of current diagnostic criteria. As the status of MMD is judged solely by the clinical diagnostic criteria, cases with stenosis only or unilateral MMD are eliminated and treated as “unaffected”, thereby rejecting the autosomal dominant mode of inheritance [31, 33]. Given that more than 80 % of Japanese patients with MMD are carriers of *RNF213* R4810K, many researchers are skeptical about such versatility of genetic loci (3p24–p26 and 8q23) in Japanese pedigrees. In earlier studies, the dogmatic application of clinical diagnosis elicited the genetic problem known as “skipping of generations”. For example, when a grandparent and grandchild are affected with MMD but the grandparent’s daughter, i.e., the mother, only has stenosis, the “skipping generation phenomenon” occurs. Several examples can be found in the study by Liu et al. [12] (Fig. 1).

To overcome these genetic irregularities, Mineharu et al. [35] conducted a genome-wide linkage analysis by introducing a “carrier state”, which widened the clinical spectrum and included phenotypes, such as stenosis, unilateral cases, or the absence of abnormalities (Fig. 1). They analyzed 15 three-generation pedigrees and obtained a single and strong linkage signal at 17q25.3 (LOD score 8.11, *p* = 3.4 × 10^−6^)with an autosomal dominant mode of inheritance. The locus at 17q.25.3 has been confirmed repeatedly by different family sets [11–17, 36], and has led to the initial identification of the susceptibility gene, *RNF213*. However, confirmation is warranted for the other loci on 3p24–p26, 8q23, and 17q25.

## Genetics of *RNF213* mutations

### R4810K and other mutations

Our previous studies showed that in East Asia, the founder variant *RNF213* R4810K was much more frequently found in MMD patients (Japanese, 90.1 %; Korean, 78.9 %; Chinese, 23.1 %) than the general population (Japanese, 2.5 %; Korean, 2.7 %; Chinese, 0.9 %) [12, 18]. Following on from these studies, several groups also identified *RNF213* R4810K in MMD patients from Taiwanese, Indian, Bangladeshi, and Filipino populations [14, 15, 37]. *RNF213* R4810K was found to be absent from control individuals as well as Caucasian MMD cases [12, 15], which may explain their lower incidence of MMD. Indeed, the MMD incidence in Caucasians was estimated to be one-tenth of that in the Japanese population [7, 38].

**Fig. 2:**
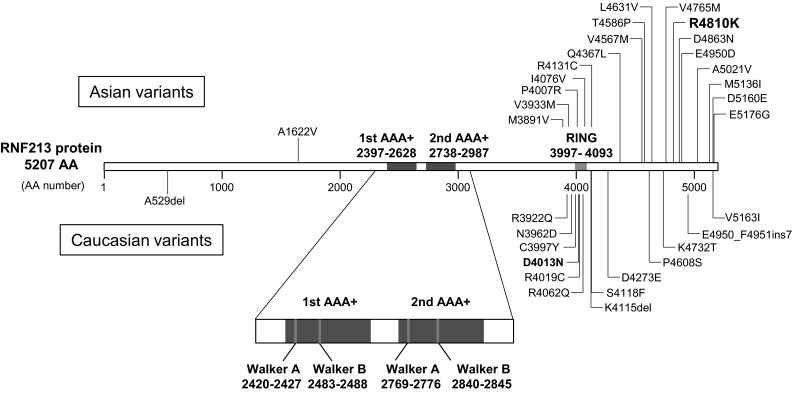
Variants shown are described previously [11, 12, 14, 15, 37, 39] (see details in Table 2). Variants in Asian and Caucasian patients are shown above and below the protein, respectively. The domain structure was based on [19]. *AA* amino acid, *AAA+* ATPase associated with diverse cellular activities domain, *RING* ring finger domain

Many non-R4810K mutations in *RNF213* have, however, been identified in both Asian and Caucasian MMD cases (Fig. 2; Table 2) [11, 12, 14, 15, 37, 39]. These mutations have two characteristics: (1) they cluster at the C-terminal portion of RNF213, and (2) they do not fall into the category of null mutations resulting in a loss-of-function (nonsense or frame-shift mutations). Almost all *RNF213* mutations, including R4810K (30 out of 32, expect for A529del and A1622V), are located within exons 41–68 (NM_001256071.2), corresponding to the region from the RING finger domain to the C-terminus of the RNF213 protein. Additionally, all 32 mutations are missense, in-frame deletions (A529del and K4115del), or in-frame insertions (E4950_F4951ins7). This suggests that the mutations have a dominant negative or gain-of-function effect. Indeed, mutations in the C-terminal portion of RNF213 would be predicted to cause functional alterations of the protein, which is more likely to be linked to a dominant negative or gain-of-function than a loss-of-function mechanism.

**Table 2:** *RNF213* mutations other than R4810K in MMD patients

Mutation	Ethnicity	References
A1622V	Asian	[37]
M3891V	Asian	[11]
V3933M	Asian	[37]
P4007R	Asian	[14]
I4076V	Asian	[15]
R4131C	Asian	[37]
Q4367L	Asian	[14]
V4567M	Asian	[11]
T4586P	Asian	[14]
L4631V	Asian	[14]
V4765M	Asian	[11]
D4863N	Asian	[12]
E4950D	Asian	[12, 14]
A5021V	Asian	[12, 14]
M5136I	Asian	[14]
D5160E	Asian	[12]
E5176G	Asian	[12]
A529del	Caucasian	[15]
R3922Q	Caucasian	[15]
N3962D	Caucasian	[12]
D4013N	Caucasian	[12, 15]
R4019C	Caucasian	[15]
R4062Q	Caucasian	[12]
C3997Y	Caucasian	[15]
K4115del	Caucasian	[15]
S4118F	Caucasian	[39]
D4273E	Caucasian	[15]
P4608S	Caucasian	[12]
K4732T	Caucasian	[15]
E4950_F4951ins7	Caucasian	[15]
V5163I	Caucasian	[15]

Interestingly, five of these non-R4810K mutations are thought to be disease causing. D4013N in Caucasian patients and E4950D and A5021V in Chinese patients, originally identified by our group [12], have also been independently reported by others [14, 15]. D4013N segregation was confirmed both in European [12] and American [15] MMD pedigrees, raising the possibility that D4013N may have a founder effect in Caucasian populations worldwide. Furthermore, the two *de novo* mutations K4115del [15] and S4118F [39] have been identified in Caucasian cases. They are located in close proximity to each other, and were detected in early onset (<1-year-old) MMD patients, indicating that mutations in this region might have severe deleterious effects on RNF213 function.

### Gene dosage effects

Gene dosage effects of *RNF213* R4810K have been reported in a clinical genetics/epidemiological study and a case report by Miyatake et al. [13]. Homozygous *RNF213* R4810K (AA) carriers with MMD were observed, but homozygosity was not seen in unaffected controls. Moreover, homozygosity was also associated with an earlier age of onset and greater disease severity compared with MMD cases harboring heterozygous *RNF213* R4810K (GA) [13]. In the case report study, which described sibling MMD cases with homozygous and heterozygous *RNF213* R4810K, the age of disease onset in the homozygote sibling was earlier than that of the heterozygote sibling, and the latter developed a milder clinical course [40]. The authors, therefore, claimed that the dosage of *RNF213* R4810K alleles was strongly associated with clinical phenotype, even in family members sharing a similar genetic background. However, we have observed homozygous *RNF213* R4810K carriers in an unaffected control population [18, 41], and also found sibling MMD cases, including identical twins, with the same dosage of *RNF213* R4810K alleles but discordant phenotypes [12]. Therefore, it appears that heterogeneity of the MMD phenotype cannot be explained solely by gene dosage effects; indeed, environmental factors may play a critical role in phenotype variation.

## Molecular characterization of RNF213

### Molecular characterization of RNF213 as an AAA+ ATPAse (ATPase associated with diverse cellular activities)

The full-length cDNA of *RNF213* was first cloned by Liu et al. [12]. It was found to code for a relatively large protein which functions both as an AAA+ ATPase and an E3 ligase (Fig. 2).

Various cell functions are mediated by AAA + ATPases, including membrane fusion/transport (NSF/Sec18p), proteolysis (ClpA), heat shock protein and protease Hsp78), motors (dyneins), protein disaggregation/refolding (Shp104/Hsp78/ClpB), DNA recombination/repair (RuvB, Rad17, Rfc2-5), and mitosis/meiosis (Cdc48p, Katanin) [42]. Morito et al. [19] demonstrated that RNF213 has two AAA+ modules and takes a hexamer form. Oligomerization is initiated by ATP binding in the Walker A motif of the first AAA+ module. This oligomer complex is then relaxed after ATP hydroxylation by the Walker B motif of the second AAA+. The cyclicity of ATP binding and ATP hydrolysis is required to generate a moving action for many AAA+ ATPases [42], which convert the chemical energy of ATP to physical energy (for example dyneins), but the role of Walker A and B motifs in maintaining ATP cyclicity is unknown.

Several diseases are known to be caused by AAA+ ATPase dysfunction, for example, *PEX1*/*PEX6* mutations cause multiple organ degeneration such as Zellweger syndrome [43, 44], while mutations in *Cdc48* cause amyotrophic lateral sclerosis [45, 46]. MMD is the only cerebrovascular or cardiovascular disease known to be associated with an AAA+ ATPase.

As RNF213 also has E3 ligase activity [12], it may additionally play a role in protein degradation or signaling processes. However, the complete physiological functions of RNF213 remain unknown as no investigations have been made into its dual AAA+ ATPase and E3 ligase activities, and its cofactors have not yet been identified.

### Interferons as natural regulators of RNF213 expression

MMD patients have been shown to have elevated levels of several growth factors in their cerebrospinal fluid, including basic fibroblast growth factor [47], transforming growth factor-β [48], platelet-derived growth factor [49], hepatocyte growth factor [50], and an uncharacterized 4473 Da peptide [51]. Recently, two groups have independently found that RNF213 is induced by interferons (IFNs) [21, 52].

Kobayashi et al. [21] demonstrated that IFNβ and IFNγ induce *RNF213* transcription in an endothelial cell (EC)-specific manner. This induction is mediated by the STAT box in the *RNF213* promoter region. Ohkubo et al. [52] also found that IFNγ and tumor necrosis factor-α synergistically activate *RNF213* transcription both in vitro and in vivo. They found that the AKT and PKR pathways contribute to the up-regulation of RNF213, although it remains to be determined what form of cellular signaling these are involved in. Further studies are needed to elucidate the complete signaling pathways associated with RNF213.

## Lowered angiogenicity of endothelial cells (ECs) as a pathological effect of *RNF213* R4810K

Kim et al. [53] reported that circulating endothelial progenitor cells obtained from patients with MMD are defective in angiogenic functions, as judged by the tube formation assay. This observation was unexpected because moyamoya vessels were thought to represent a hyperangiogenic phenomenon. This finding stimulated the following studies:

### ECs derived from MMD patient iPSCs show unique EC-specific gene expression profiles

To obtain an MMD disease model, iPSCs were established from fibroblasts donated from six subjects [24]: two wild-type controls, two *RNF213* R4810K heterozygotes (one affected and the other not affected with MMD), and two patients homozygous for *RNF213* R4810K. iPSC ECs (iPSECs) were differentiated from iPSCs, and those derived from heterozygotes or homozygotes showed significantly decreased angiogenic activities compared with control iPSECs in accordance with the observation of Kim et al. [53]. In parallel, features of lowered angiogenic activity were recapitulated in human umbilical venous endothelial cells (HUVECs) overexpressing *RNF213* R4810K, but not in those overexpressing WT RNF213. These authors also conducted expression array analyses in fibroblasts and counterpart iPSECs from the same donors. They observed differential expression profiles of mRNAs in iPSECs derived from controls and carriers of *RNF213* R4810K, but none in the fibroblasts from the same donors. A total of 121 genes were down-regulated (Supplemental Table 1) and 36 genes were up-regulated (Supplemental Table 2) [24]. These expression profile differences were considered to be functionally related to the lowered angiogenic activities of ECs. These observations strongly indicated that differentiation from stem cells (i.e., iPSCs) to ECs induced a change of the gene expression profile by RNF213 R4810K.

Attention was focused on cell cycle-associated genes (Supplemental Table 1, asterisks), because they were enriched by gene ontology category analysis as down-regulated in iPSECs from *RNF213* R4810K carriers. The expression of one of these genes, the key mitotic player *Securin* (*PTTG1*), which activates angiogenesis [54], was investigated in HUVECs and shown to be inhibited by *RNF213* R4810K overexpression [24]. RNA silencing of *Securin* in HUVECs and wild-type iPSECs was found to inhibit angiogenesis, indicating that RNF213 R4810K lowers angiogenesis, at least in part, by the down-regulation of *Securin*. As this work only focused on a single gene out of the 128 identified, the biological implication of the expression profile differences found in iPSECs requires further investigation.

Tube formation, a comprehensive measure of angiogenic activity, is affected by various factors, such as EC proliferation rates and maturity [55]. As the overexpression of RNF213 R4810K inhibited HUVEC proliferation, Hitomi et al. [56] further investigated the effects of RNF213 R4810K on the cell cycle using HeLa cells, fibroblasts, and iPSECs. They found that overexpression of RNF213 R4810K, but not WT RNF213, delayed mitosis in HeLa cells, and that this was associated with abnormal mobilization of the metaphase–anaphase spindle checkpoint protein, mitotic arrest deficient 2 (MAD2). This abnormal mobilization was also seen in patient fibroblasts. Furthermore, both WT and mutant RNF213 could be co-immunoprecipitated with MAD2. Finally, iPSECs from MMD patients had higher mitotic failure rates than those from controls.

Collectively lowered angiogenic activity in vitro data suggest that RNF213 R4810K acts on EC signal production and proliferation/cell cycle. Deleterious cell proliferation/cell cycles are mediated by Securin and/or MAD2, which cross-talk with mutant, and probably WT, RNF213. As cell cycle abnormality is a common denominator for some monogenic diseases, such as Schimke immuno-osseous dysplasia, MOPDII, or Seckel syndrome (Table 1), further studies are warranted to explore this.

### Lowered angiogenicity of RNF213 R4810K as an AAA + ATPase

**Fig. 3:**
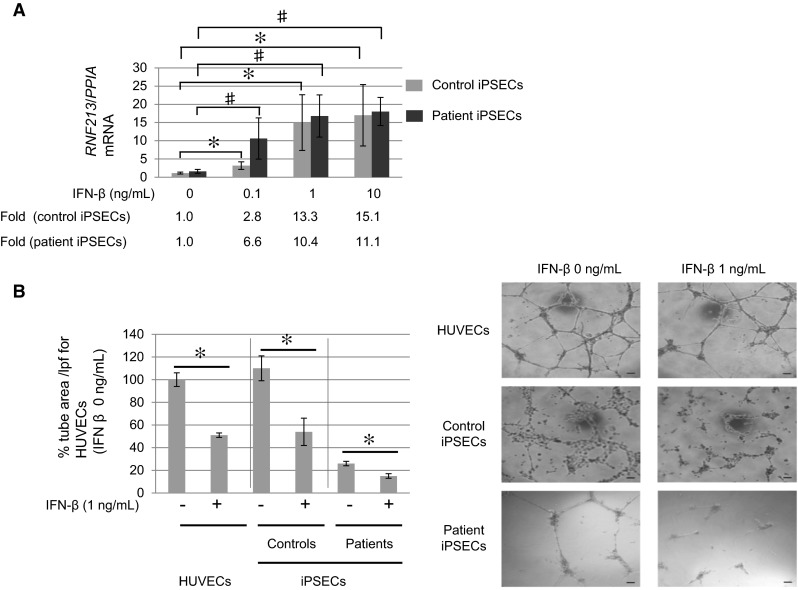
Inhibition of angiogenesis by INFβ and lowered angiogenic activity of iPSECs established from controls and patients. iPSCs were established from controls and patients with MMD. Mature iPSECs were developed from iPSCs as reported by Hitomi et al. [24]. **a** Treatment with INFβ induced mRNA of RNF213 significantly. **b** Tube formation was lowered in patients. Treatment with INFβ inhibited tube formation for iPSEECs from patients. Cited from Kobayashi et al. [21]

Kobayashi et al. [21] investigated the effects of RNF213 R4810K induction on angiogenic activity, as measured by tube formation and by the migration assay. They confirmed that treatment with IFNβ, a cytokine that inhibits both angiogenesis and arteriogenesis [57, 58], inhibited angiogenesis in iPSECs (Fig. 3). This reduced angiogenesis could be rescued either by STAT box (Signal Transduction and Transcription) or RNF213 depletion in HUVECs. This led to the conclusion that the reduced anti-angiogenic activity of IFNβ is partially mediated by RNF213, which acts as a mediator downstream of the IFNβ signaling pathway. They also confirmed that overexpression of RNF213 R4810K, but not WT RNF213, can recapture the reduced angiogenicity induced by IFNβ, suggesting that RNF213 R4810K overexpression mimics IFNβ action.

Morito et al. [19] demonstrated that disruption of Walker A or B motifs on the first or second AAA + modules decreases ATPase activity. However, while both motifs are necessary to maintain the oligomeric state, the Walker B motif has little impact on oligomerization. Furthermore, Morito et al. demonstrated that RNF213 R4810K forms a hexamer complex similar to the WT protein. Kobayashi et al. [21] further investigated the AAA+ ATPase mechanism by overexpressing various mutants in HUVECs: vector, RNF213 WT, RNF213 R4810K, a mutation of RNF213 Walker B motif (E2488Q) on the first AAA+ module (RNF213 WEQ), which disrupts ATP hydrolysis activity, and RNF213 first AAA+ module deletion mutant (RNF213 ΔAAA). They found that RNF213 R4810K and RNF213 WEQ, but neither RNF213 WT nor RNF213 ΔAAA, inhibited angiogenesis compared with the vector alone. They further showed that the ATPase activity was decreased in HUVECs transfected with RNF213 R4810K, RNF213 WEQ, and RNF213 ΔAAA. These results indicate that RNF213 R4810K is a molecular mimic of RNF213 WEQ. This also suggested that the Walker B motif in the first AAA+ module is functionally important in manifesting the function of ECs. A possible explanation for this is that disruption of the RNF213 first B motif disrupts ATP hydrolysis cyclicity, thereby inhibiting angiogenesis. As RNF213 R4810K is considered to have a similar mode of action to RNF213 WEQ, we speculate that it impairs the ATP hydrolysis cycle in the same way as the Walker B mutation.

### RNF213 R4810K showed a reduced angiogenesis response to hypoxia in vivo

Kobayashi et al. [21] also focused their attention on the effects of RNF213 R4810K on angiogenesis after hypoxia exposure in vivo. They developed transgenic mouse (Tg) strains overexpressing *RNF213* R4757K (the mouse homolog of human R4810K) in ECs or vascular smooth muscle cells (SMCs).

**Fig. 4:**
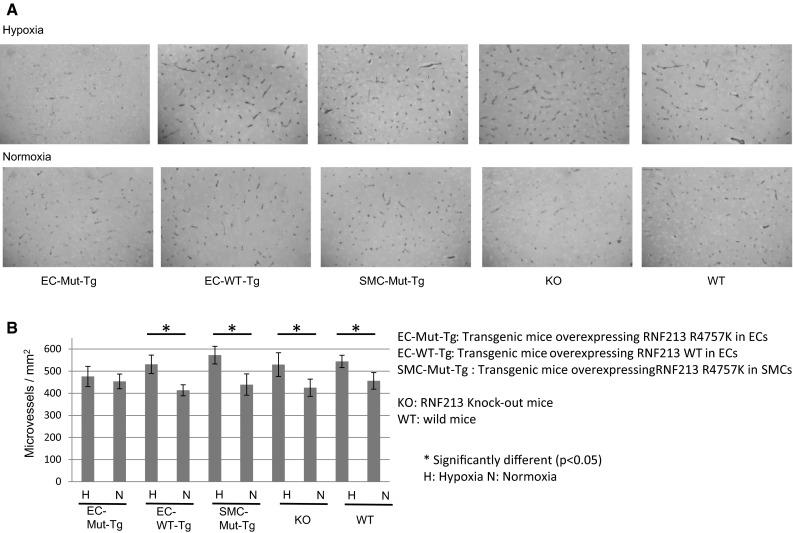
Lowered adaptive cerebral angiogenesis after exposure to hypoxia in transgenic mice expressing RNF213 R4757K in endothelial cells. **a** Several *lines* of transgenic or knockout or wild-type mice were exposed to hypoxia for 2 weeks at 8 % oxygen. Cerebral angiogenesis was evaluated by immunostaining Glut4. **b** Adaptive cerebral angiogenesis was abolished in transgenic mice overexpressing RNF213 R4757K (Human allelic ortholog of R4810K) in ECs, while in other mice adaptive angiogenesis was observed. Cited from Kobayashi et al. [21]

Hypoxia is known to induce angiogenesis in the cerebrum [59]. Mice were exposed to hypoxia (8 % O_2_) for 2 weeks from 3 weeks of age. Angiogenesis was found to be specifically reduced in Tg-ECs overexpressing RNF213 R4757K compared with other strains, i.e., Tg-SMCs overexpressing RNF213 R4757K or Tg WT RNF213 overexpressing RNF213 wild type specifically in ECs or RNF213 knock-out (KO) or WT mice (Fig. 4). The authors could recapture the lowered angiogenicity of Tg ECs in vivo, but magnetic resonance imaging failed to identify stenosis in the cerebral arteries or infarction.

As lowered angiogenesis induced by RNF213 R4810K (R4757K in the mouse) observed in the in vitro ECs (HUVEC or iPSECs) could be successfully recaptured in the Tg mouse model overexpressing RNF213 R4757K, this suggested that ECs of *RNF213* R4810K carriers may have a lowered angiogenicity and be particularly susceptible to hypoxia.

### Other relevant studies

RNF213 KO mice were established by Kobayashi et al. [20], but did not induce abnormalities in the cardiovascular system. The effects of RNF213 ablation of diabetic progression were studied in the Akita mouse [60] which develops diabetes through an unfolded protein response of insulin 2. The authors tested whether RNF213 ablation (KO) influenced the development of diabetes and intracranial arteries around the circle of Willis. Although no stenosis was detected in the cerebral arteries of the RNF213 KO mouse, significant alleviation of endoplasmic reticulum (ER) stress was observed in pancreatic beta cells. Because ER stress enhances protein degradation and consequently depletes insulin levels in the Akita mouse, the authors speculated that RNF213 is involved in protein degradation as an E3 ligase in the proteasome.

Sonobe et al. [61] investigated the effects of RNF213 KO on vascular anatomy. They investigated cranial arteries using high-resolution magnetic resonance angiography, but found no abnormalities. They also investigated the effects on vascular remodeling after ligation of the carotid artery, but could not replicate the stenotic region, a hallmark of MMD. Conversely, Ito et al. [23] recently reported the recovery of blood flow after hind limb ischemia by femoral artery ligation in RNF213 KO mice. Recoveries were enhanced in RNF213 KO mice compared with WT counterparts. Although RNF213 KO animal models have yielded conflicting results in the cerebrum and hind limbs, Fujimura et al. [62] speculated that RNF213 influences vascular remodeling in chronic ischemia.

### Inconsistencies necessitate additional experiments

Liu et al. [12] found that the inhibition of RNF213 expression in zebrafish induces abnormal arteriogenesis, but this is not observed in KO mouse models [20–22] despite the enhanced post-ischemic angiogenesis seen in the KO mouse [23]. This finding may be physiologically compatible with lowered angiogenesis in the cerebrum after hypoxic exposure in EC-specific R4757K Tg mice [21]; thus, the overexpression of RNF213 R4758K in ECs inhibits angiogenesis and conversely RNF213 depletion enhances angiogenesis.

Further discrepancies are noted between the observed inhibition of angiogenesis following RNF213 R4810K overexpression [21] and that in HUVECs following RNF213 depletion [52]. These differences are associated with the controversies in the reported *RNF213* R4810K genetic mechanisms, involving loss-of-function, gain-of-function [52, 62], and dominant negative [21]. Alternatively, they could reflect species differences in innate immunity, e.g., of zebrafish and mice [63, 64], and further studies are needed to resolve these discrepancies.

## Hypothetical pathological roles of RNF213 R4810K in MMD

### Three major abnormalities: ECs, SMCs, and hemostasis

**Fig. 5:**
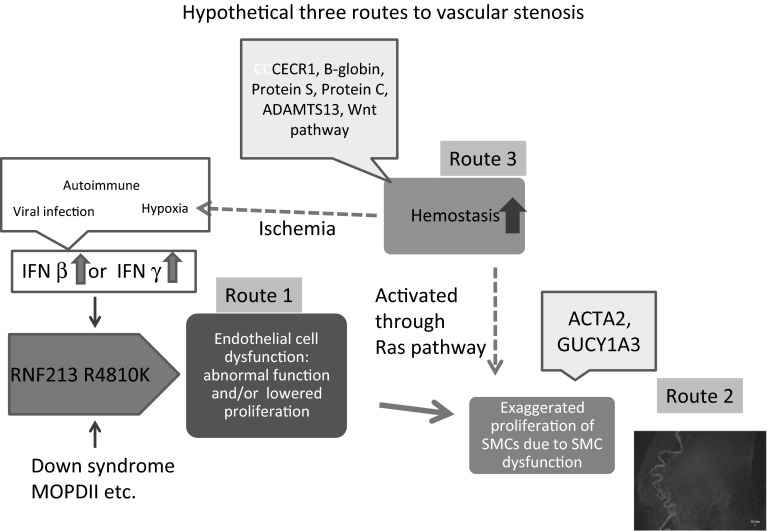
Three-route model of the hypothetical molecular pathology of moyamoya disease/syndrome. The model assumes that any of three independent abnormalities, endothelial dysfunction, smooth muscle cell dysfunction, and abnormal hemostasis, can lead to exaggerated proliferation of SMCs. Each abnormality can result in vascular stenosis. *RNF213* R4810K is the major detrimental factor that elicits endothelial cell dysfunction. Pro-inflammatory signals such as IFNs can activate the transcription of *RNF213*

Several monogenic diseases have been reported to be complicated by MMD (Table 1). As various biological processes are involved in these diseases, including signal transduction, chromatin remodeling/DNA repair, DNA repair/angiogenesis, inflammation, vascular smooth muscle cell dysfunction, and coagulopathy, the pathological process of MMD cannot be explained in a consolidated signaling pathway. However, the diseases can be classified into three major abnormalities: (1) impaired functions of ECs, (2) SMC dysfunction, and (3) hemostasis abnormalities. For simplicity, we would like to propose an intuitive working hypothesis based on Table 1 and recent findings on RNF213. For analogy, we call this a three-route model (Fig. 5), in which MMD can occur through three different routes. These routes lead to the common outcome of SMC proliferation.

In the first route, RNF213 functions as a key mediator in ECs. Given that Type I IFN overproduction (Aicardi–Goutieres syndrome) [65] is complicated by MMD and RNF213 is highly activated by IFNs [21, 52], pro-inflammatory signals enhance IFN overproduction, which then activates *RNF213* transcription. It should also be noted that pro-inflammatory signals can be induced by viral infections or by damaged, unrepaired DNA [65]. Amplified pro-inflammatory signals can lead to thrombosis, as seen in Sneddon’s syndrome [66–69].

In the second route, SMC dysfunction, which leads to exaggerated SMC proliferation, is a major outcome. Alpha-actin-2 (*ACTA2*) and guanylate cyclase 1 (*GUCY1A3*) are known to promote vascular SMC proliferation and induce MMD [70, 71]. While *ACTA2* mutation causes moyamoya syndrome with thoracic aortic aneurysm and dissection by the mode of autosomal dominant, *GUCY1A3* induced moyamoya syndrome with achalasia in the mode of autosomal recessive. It is of particular interest that *GUCY1A3* encodes the major nitric oxide receptor. In Alagille syndrome (involving the Ras pathway), the impaired differentiation of both ECs and SMCs occurs [72].

The last route is associated with hemostasis. Several diseases in this category are known to induce hemostatic abnormalities, including Sickle-cell disease [73], mutations of protein S and protein C [74, 75], thrombotic thrombocytopenic purpura [76], and Noonan syndrome [77]. Furthermore, genes in the Ras signaling pathway [78] and Wnt signaling pathway [79] influence platelet activation. Activated thrombi formation results in ischemia and thereby causing hypoxia. In addition, Ras pathways may also trigger vascular inflammation [80] or SMC dysfunction [81] in a direct way.

### Hypoxia, vascular injury, or chronic inflammation generate pro-inflammatory signals: stimulators of stenosis (neointimal formation)

It has been consistently demonstrated that RNF213 R4810K lowers angiogenic activities in ECs [21, 24, 53], but it remains unclear how this leads to stenosis (neointimal formation). This may be answered by examining pro-inflammatory signals, such as those involved in the JAK-STAT pathway.

Hypoxia [82], vascular injury [83], and chronic inflammation accompanied by elevated Type I IFNs [84] are known to activate EC mobilization for angiogenesis, which in turn leads to the production of adhesion molecules, cytokines, and chemokines. These pro-inflammatory signals stimulate SMC proliferation, migration, and secretion of extracellular matrix, causing neointimal formation [85]. Recently, IFN regulatory factors, activated by IFNα/β, were reported to modulate neointimal formation with sirtuin (SIRT)1 [86]. Given that RNF213 R4810K is a mimic of IFNβ, it may amplify the effects of IFNα/β, thereby magnifying neointimal formation by perturbing the IRF9/SIRT1 axis. The investigation of cytokine signaling in cells expressing RNF213 R4810K is expected to provide answers to several of these pending questions.

## Future public health contributions to MMD

Since *RNF213* was identified as a susceptible gene for MMD and as a founder mutation carried by 15 million people from the East Asian population [18], it has emerged as a key player in vascular disease. However, recent progress has also resulted in some unanswered questions, such as how can RNF213 R4810K describe the entire spectrum of the diseases associated with MMD? What is the health risk to *RNF213* R4810K carriers? Can environmental factors explain the observed low penetrance of one in 150 carriers developing MMD?

Although in vitro and in vivo experimental approaches are expected to address many of these unresolved biomedical questions, well-designed human epidemiological studies are also essential. Given the large number of *RNF213* R4810K carriers in the general population, there is an urgent need to evaluate their health risks. In parallel, ethical issues should be taken into account to avoid genetic stigmatization of these carriers.

### A broader definition of MMD

The current diagnosis of MMD is based on the definition of the angiographic appearance of moyamoya vessels. However, *RNF213* R410K genetics has unveiled different stages of disease progression. Given that 80 % of MMD cases are carriers of *RNF213* R4810K, a definition of MMD based on this seems broader than one based on angiography. Another enigma is the difference between MMD and moyamoya syndrome. Chong et al. [87] recently reported a Down syndrome case with MMD, who is a carrier of *RNF213* R4810K. This indicates that the interaction of *RNF213* R4810K with other genes can lead to the manifestation of MMD. Within this context, the relationship between MMD syndrome and RNF213 should be examined and thereby definition of MMD being expanded. Indeed, a broader diagnostic criterion based on *RNF213* is needed to illustrate the entire spectrum of MMD, as well as to delineate the natural course of carriers of *RNF213* mutations in an epidemiological study.

### Health risks associated with *RNF213* R4810K

Prevalence of stenotic lesions or MMD was significantly higher (larger than 20 %) in the carriers of R4810K, if the carrier has family history of MMD [12]. The high risk among carriers in familial MMD shows a sharp contrast with the low risk of carriers in general population. Thus, carriers in the familial MMD is worthy for follow-up to ensure the early intervention.

Recently, Koizumi et al. [41] conducted a genetic epidemiological study with a case–control design (*N* = 4308) to investigate the association of *RNF213* R4810K with blood pressure in the general Japanese population. They found 60 carriers (1.4 %). Regression analysis adjusted for age, sex, and body mass index based on the additive model demonstrated significant association with systolic blood pressure (mmHg/allele): β (Standard errors) 8.9 (2.0) (*p* = 10^−5^). In contrast, diastolic blood pressure did not show the association. Those data strongly indicate that RNF213 R4810K is a risk factor of blood pressure in free-living carriers in general population.

Monogenic diseases stochastically associated with MMD are often accompanied by coronary heart diseases (CHD) (*BRCC3* [88] and *ACTA2* [70]). Similarly, several case studies have reported the association between MMD and CHD [89–92]. Recently, Nam et al. [92] found that 4.6 % of 456 MMD patients were affected with CHD. Because these patients were young and lacked CHD risk factors, this suggests that CHD may be accelerated by the presence of *RNF213* R4810K.

In early pathological studies [93–95], arterial stenosis was found to occur systematically, not only in the intracranial arteries but also in coronary, pulmonary, renal, and pancreatic arteries. Therefore, *RNF213* R4810K carriers may have ischemic damage in these organs. These findings collectively imply that stenotic regions occur in various arteries and suggest the existence of both cardio- and cerebrovascular risks. Future large-scale genetic cohort studies should evaluate the risk of *RNF213* R4810K on health outcomes of cardio- and cerebrovascular diseases, such as ischemic stroke, hemorrhagic stroke, myocardial infarction, and hypertension.

## Environmental factors to explain low penetrance

The total number of registered MMD patients in 2012 was 15,177 in Japan (http://www.nanbyou.or.jp/entry/3664, Aug 5, 2015). Assuming that 80 % of these patients are carriers, the prevalence of MMD is 10^−4^. Carriers are estimated to be 2 % of the general population, resulting in only one out of ~150 carriers developing MMD. Therefore, another factor is needed to explain such a 1/150 low penetrance.

Kaku et al. [96] reported that vascular constrictive changes of affected arteries occur in MMD in comparison with other steno-occlusive diseases. It is uncertain whether such changes represent anatomical abnormalities involving narrowing of the cavernous sinus. Given that these are rare, they may increase the risk of MMD for *RNF213* R4810K carriers. The constrictive remodeling hypothesis can be tested in animal models by introducing stenosis in the carotid artery. However, to date, ligation of the carotid artery has failed to replicate intimal hyperplasia when applied to the RNF213 ablation mouse [61]. Further studies are, therefore, warranted to test this hypothesis.

Inflammation is another possibility to explain such a low penetrance. Kobayashi et al. [21] and Ohkubo et al. [52] recently demonstrated that IFNs activate *RNF213* transcription, so inflammation may induce MMD in association with *RNF213* R4810K.

Yamashita et al. [25] reported that thrombi formation predominantly occurs in intracranial arteries of MMD patients, while Ikeda confirmed its presence in systemic arties [93]. Thrombi formation is often an opportunistic event precipitated by environmental factors, and we, therefore, speculate that this partially explains the 1/150 gap.

At present, it is highly probable that inflammatory signals trigger MMD. However, there has been no epidemiological evidence on the association of infection histories or other life-style or behavior factors with MMD in the carriers. Epidemiological evidence obtained through cohort studies focusing on carriers is deficient at present and is needed.

### Therapeutic approach

Several studies have shown that the ablation of RNF213 does not cause deleterious effects on angiogenesis, except in zebrafish [12, 20, 61], suggesting that it might not affect mammalian species. A promising hypothesis is that *RNF213* R4810K causes MMD by a dominant negative or gain-of-function mechanism. If this is the case, a pharmacological antagonist that inhibits ATP binding to the Walker A motif would be a suitable candidate as a drug target.

### Ethical issues

*RNF213* R4810K carriers have a very high prevalence in Japan and Korea (1–2 %) and are extremely likely to develop MMD. At present, however, there are insufficient data to predict the health risk of the carriers, except for subjects of familial MMD. It is, therefore, important to obtain carrier data and to elucidate the MMD risk attributable to *RNF213* R4810K. In parallel, public health researchers should collaborate with genetic counsellors to facilitate genetic risk communication, not only to carriers, but also to society to avoid the social discrimination of carriers. A great deal of uncertainty currently surrounds application of genetic testing to the general population; indeed, it may have no benefit for the general population and only limited benefit for unaffected members in familial cases with MMD.

## Conclusions

MMD was first described in 1957 by Takeuchi and Shimizu [97]. Although genetic factors had long been speculated, the angiographic definition of the MMD likely misled its genetic analysis. Recently, *RNF213* R4810K was identified as the major susceptibility gene [10–12], and full-length cDNA cloning, iPS technology, and animal models have enabled the pathological roles of *RNF213* R4810K to be investigated. Systemic biomedical and genetic epidemiology studies with specific emphasis on carriers will provide a deep understanding not only of MMD but also of associated health and environmental risk factors. Furthermore, such research will lead to a novel disease definition than the present one, and pave the way for new preventive strategies for cerebrovascular diseases, especially those in children.

## Electronic supplementary material

Supplementary material 1 (DOCX 28 kb)
