# Supplementary material for: A new horizon of moyamoya disease and associated health risks explored through RNF213
Source: Environ Health Prev Med. 2015 Dec 10;21(2):55–70. doi: 10.1007/s12199-015-0498-7 (PMC4771639; doi:10.1007/s12199-015-0498-7)
Supplement: Supplementary file 1 — Supplementary material 1 (DOCX 28 kb) [file 12199_2015_498_MOESM1_ESM.docx]

**Supplemental Table 1** Genes down-regulated significantly (*p*<0.01) more than three-fold in endothelial cells derived from MMD patient iPS cells compared with controls [24]

| **Order of gene** | **Gene  Symbol** | **Gene  Description** | **Fold  Change: AA and GA vs GG** |
| --- | --- | --- | --- |
| 1 | NPY | neuropeptide Y | 16.83 |
| 2 | MYOCD | myocardin | 11.82 |
| 3 | GPC3 | glypican 3 | 10.97 |
| 4 | PROM1 | prominin 1 | 10.80 |
| 5 | P2RY14 | purinergic receptor P2Y, G-protein coupled, 14 | 10.57 |
| 6 | CLDN11 | claudin 11 | 9.99 |
| 7 | RGS13 | regulator of G-protein signaling 13 | 9.25 |
| 8 | EDNRB | endothelin receptor type B | 6.87 |
| 9 | LRRC17 | leucine rich repeat containing 17 | 6.73 |
| 10 | SERPINF1 | serpin peptidase inhibitor, clade F (alpha-2 antiplasmin, pigment epithelium derived factor), member 1 | 6.51 |
| 11 | ACTG2 | actin, gamma 2, smooth muscle, enteric | 5.89 |
| 12 | DLK1 | delta-like 1 homolog (Drosophila) | 5.89 |
| 13 | CDC20* | cell division cycle 20 homolog (S. cerevisiae) | 5.81 |
| 14 | TOP2A* | topoisomerase (DNA) II alpha 170kDa | 5.61 |
| 15 | SLC13A5 | solute carrier family 13 (sodium-dependent citrate transporter), member 5 | 5.42 |
| 16 | KIF20A\|CDC23* | kinesin family member 20A \| cell division cycle 23 homolog (S. cerevisiae) | 5.28 |
| 17 | HIST1H3B* | histone cluster 1, H3b | 5.27 |
| 18 | ASPM | asp (abnormal spindle) homolog, microcephaly associated (Drosophila) | 5.26 |
| 19 | PLK1 | polo-like kinase 1 | 5.16 |
| 20 | HIST1H1B* | histone cluster 1, H1b | 4.73 |
| 21 | CD200 | OX-2 membrane glycoprotein | 4.72 |
| 22 | ANLN | anillin, actin binding protein | 4.72 |
| 23 | NUF2 | NDC80 kinetochore complex component | 4.60 |
| 24 | LGI1 | leucine-rich, glioma inactivated 1 | 4.56 |
| 25 | LGR5 | leucine-rich repeat-containing G protein-coupled receptor 5 | 4.56 |
| 26 | IFITM1 | interferon induced transmembrane protein 1 (9-27) | 4.47 |
| 27 | DLGAP5 | discs, large (Drosophila) homolog-associated protein 5 | 4.46 |
| 28 | CCNB2* | cyclin B2 | 4.39 |
| 29 | PBK | PDZ binding kinase | 4.37 |
| 30 | NCAPG\|LCORL | non-SMC condensin I complex, subunit G \| ligand dependent nuclear receptor corepressor-like | 4.34 |
| 31 | MKI67 | antigen identified by monoclonal antibody Ki-67 | 4.33 |
| 32 | BUB1* | budding uninhibited by benzimidazoles 1 homolog (yeast) | 4.28 |
| 33 | CDKN3* | cyclin-dependent kinase inhibitor 3 | 4.28 |
| 34 | PRC1* | protein regulator of cytokinesis 1 | 4.27 |
| 35 | ITGA8 | integrin, alpha 8 | 4.27 |
| 36 | CCBE1 | collagen and calcium binding EGF domains 1 | 4.25 |
| 37 | TPX2 | microtubule-associated | 4.21 |
| 38 | NUSAP1 | nucleolar and spindle associated protein 1 | 4.18 |
| 39 | SKA3\|MRP63* | spindle and kinetochore associated complex subunit 3 \| mitochondrial ribosomal protein 63 | 4.17 |
| 40 | PARM1 | prostate androgen-regulated mucin-like protein 1 | 4.17 |
| 41 | COL3A1 | collagen, type III, alpha 1 | 4.13 |
| 42 | CKAP2L | cytoskeleton associated protein 2-like | 4.13 |
| 43 | SRGN | serglycin | 4.09 |
| 44 | CENPF* | centromere protein F, 350/400kDa (mitosin) | 4.08 |
| 45 | CDK1* | cyclin-dependent kinase 1 | 4.08 |
| 46 | COL6A3 | collagen, type VI, alpha 3 | 4.06 |
| 47 | KIF11 | kinesin family member 11 | 3.98 |
| 48 | DTL | denticleless homolog (Drosophila) | 3.96 |
| 49 | ST6GAL2 | ST6 beta-galactosamide alpha-2,6-sialyltranferase 2 | 3.95 |
| 50 | KIAA0101\|CSNK1G1 | KIAA0101 \| casein kinase 1, gamma 1 | 3.93 |
| 51 | BUB1B\|PAK6* | budding uninhibited by benzimidazoles 1 homolog beta (yeast) \| p21 protein (Cdc42/Rac)-activated kinase 6 | 3.93 |
| 52 | CEP55 | centrosomal protein 55kDa | 3.91 |
| 53 | HIST1H3J | histone cluster 1, H3j | 3.89 |
| 54 | CCNA2* | cyclin A2 | 3.83 |
| 55 | CASC5 | cancer susceptibility candidate 5 | 3.79 |
| 56 | ARHGAP11A | Rho GTPase activating protein 11A | 3.76 |
| 57 | STXBP5L | syntaxin binding protein 5-like | 3.76 |
| 58 | NCAPH* | non-SMC condensin I complex, subunit H | 3.75 |
| 59 | POU5F1\|POU5F1P3\|POU5F1P4\|POU5F1B | POU class 5 homeobox 1 \| POU class 5 homeobox 1 pseudogene 3 \| POU class 5 homeobox 1 pseudogene 4 \| POU class 5 homeobox 1B | 3.73 |
| 60 | FRZB | frizzled-related protein | 3.72 |
| 61 | RNU6-336P |  | 3.69 |
| 62 | HIST2H2AB | histone cluster 2, H2ab | 3.68 |
| 63 | DEPDC1 | DEP domain containing 1 | 3.62 |
| 64 | PCDH18 | protocadherin 18 | 3.60 |
| 65 | ELOVL2 | elongation of very long chain fatty acids (FEN1/Elo2, SUR4/Elo3, yeast)-like 2 | 3.60 |
| 66 | SGOL1 | shugoshin-like 1 (S. pombe) | 3.58 |
| 67 | AIM1 | absent in melanoma 1 | 3.55 |
| 68 | RRM2 | ribonucleotide reductase M2 | 3.53 |
| 69 | ANK3 | ankyrin 3, node of Ranvier (ankyrin G) | 3.51 |
| 70 | FOXM1 | forkhead box M1 | 3.51 |
| 71 | KIF18A | kinesin family member 18A | 3.50 |
| 72 | WDR76 | WD repeat domain 76 | 3.47 |
| 73 | TMEM71 | transmembrane protein 71 | 3.46 |
| 74 | TTK | dual specificity protein kinase | 3.45 |
| 75 | PDE3A | phosphodiesterase 3A, cGMP-inhibited | 3.41 |
| 76 | POU5F1\|POU5F1P3\|POU5F1P4\|POU5F1B | POU class 5 homeobox 1 \| POU class 5 homeobox 1 pseudogene 3 \| POU class 5 homeobox 1 pseudogene 4 \| POU class 5 homeobox 1B | 3.41 |
| 77 | POU5F1\|POU5F1P3\|POU5F1P4\|POU5F1B | POU class 5 homeobox 1 \| POU class 5 homeobox 1 pseudogene 3 \| POU class 5 homeobox 1 pseudogene 4 \| POU class 5 homeobox 1B | 3.41 |
| 78 | KIF23 | kinesin family member 23 | 3.40 |
| 79 | RNU6-1241P |  | 3.40 |
| 80 | GINS1 | GINS complex subunit 1 (Psf1 homolog) | 3.39 |
| 81 | SLIT3 | slit homolog 3 (Drosophila) | 3.38 |
| 82 | SHISA3 | shisa homolog 3 (Xenopus laevis) | 3.37 |
| 83 | MYBL2 | v-myb myeloblastosis viral oncogene homolog (avian)-like 2 | 3.37 |
| 84 | Securin* | pituitary tumor-transforming 1 | 3.37 |
| 85 | CDCA2* | cell division cycle associated 2 | 3.36 |
| 86 | SHCBP1 | SHC SH2-domain binding protein 1 | 3.34 |
| 87 | EMP2 | epithelial membrane protein 2 | 3.34 |
| 88 | PLK4* | polo-like kinase 4 | 3.33 |
| 89 | HIST2H3D\|HIST2H3A\|HIST2H3C | histone cluster 2, H3d \| histone cluster 2, H3a \| histone cluster 2, H3c | 3.33 |
| 90 | HMMR | hyaluronan-mediated motility receptor (RHAMM) | 3.31 |
| 91 | KIF15 | kinesin family member 15 | 3.30 |
| 92 | STMN2 | stathmin-like 2 | 3.30 |
| 93 | HIST1H2BH | histone cluster 1, H2bh | 3.29 |
| 94 | HIST1H3F | histone cluster 1, H3f | 3.29 |
| 95 | NDST3 | N-deacetylase/N-sulfotransferase (heparan glucosaminyl) 3 | 3.27 |
| 96 | SCARA3 | scavenger receptor class A, member 3 | 3.27 |
| 97 | SPAG5\|SGK494 | sperm associated antigen 5 \| uncharacterized serine/threonine-protein kinase SgK494 | 3.23 |
| 98 | PLCXD3 | phosphatidylinositol-specific phospholipase C, X domain containing 3 | 3.23 |
| 99 | RGS2 | regulator of G-protein signaling 2, 24kDa | 3.23 |
| 100 | C15orf42 | chromosome 15 open reading frame 42 | 3.22 |
| 101 | ESCO2 | establishment of cohesion 1 homolog 2 (S. cerevisiae) | 3.22 |
| 102 | PRR11 | proline rich 11 | 3.21 |
| 103 | PCOLCE | procollagen C-endopeptidase enhancer | 3.16 |
| 104 | ASF1B | ASF1 anti-silencing function 1 homolog B (S. cerevisiae) | 3.14 |
| 105 | GINS2 | GINS complex subunit 2 (Psf2 homolog) | 3.14 |
| 106 | APCDD1 | adenomatosis polyposis coli down-regulated 1 | 3.13 |
| 107 | ODZ2 | odz, odd Oz/ten-m homolog 2 (Drosophila) | 3.13 |
| 108 | FAM134B | family with sequence similarity 134, member B | 3.12 |
| 109 | TNFRSF9 | tumor necrosis factor receptor superfamily, member 9 | 3.12 |
| 110 | NEIL3 | nei endonuclease VIII-like 3 (E. coli) | 3.12 |
| 111 | SKA1* | spindle and kinetochore associated complex subunit 1 | 3.10 |
| 112 | BRIP1* | BRCA1 interacting protein C-terminal helicase 1 | 3.10 |
| 113 | POU5F1B | POU class 5 homeobox 1B | 3.09 |
| 114 | NDC80 | kinetochore protein NDC80 homolog | 3.09 |
| 115 | CD180 | CD180 antigen | 3.06 |
| 116 | FGL2 | fibrinogen-like 2 | 3.06 |
| 117 | FAM111B | family with sequence similarity 111, member B | 3.04 |
| 118 | POU5F1P3 | POU class 5 homeobox 1 pseudogene 3 | 3.03 |
| 119 | HIST1H3I | histone cluster 1, H3i | 3.02 |
| 120 | SLC4A4 | solute carrier family 4, sodium bicarbonate cotransporter, member 4 | 3.01 |
| 121 | TRIL | TLR4 interactor with leucine-rich repeats | 3.01 |
| * Associated with cell proliferation and cell cycle | | | |

**Supplemental Table 2** Genes up-regulated significantly (*p*<0.01) more than three-fold in endothelial cells derived from patient iPS cells compared with controls [24]

| **Order of gene** | **Gene  Symbol** | **Gene  Description** | **Fold  Change: AA and GA vs GG** |
| --- | --- | --- | --- |
| 1 | MMP1 | matrix metallopeptidase 1 (interstitial collagenase) | 12.72 |
| 2 | ATP6V0D2 | ATPase, H+ transporting, lysosomal 38kDa, V0 subunit d2 | 12.11 |
| 3 | SERPINB4\|SERPINB3 | serpin peptidase inhibitor, clade B (ovalbumin), member 4 \| serpin peptidase inhibitor, clade B (ovalbumin), member 3 | 7.86 |
| 4 | CXCL12 | chemokine (C-X-C motif) ligand 12 | 6.07 |
| 5 | INHBA | inhibin, beta A | 6.05 |
| 6 | CDH10 | cadherin 10, type 2 (T2-cadherin) | 6.00 |
| 7 | SNORD116-21\|SNORD116@ | small nucleolar RNA, C/D box 116-21 \| small nucleolar RNA, C/D box 116 cluster | 5.71 |
| 8 | MMP10 | matrix metallopeptidase 10 (stromelysin 2) | 5.59 |
| 9 | ULBP1 | UL16 binding protein 1 | 5.57 |
| 10 | HTN1 | histatin 1 | 5.12 |
| 11 | NEFL\|LOC100129717 | neurofilament, light polypeptide \| hypothetical LOC100129717 | 4.53 |
| 12 | MYOZ2 | myozenin 2 | 4.16 |
| 13 | APLN | apelin | 4.05 |
| 14 | EDN1 | endothelin 1 | 4.04 |
| 15 | MYCT1 | myc target 1 | 4.03 |
| 16 | SHISA9 | shisa homolog 9 (Xenopus laevis) | 3.93 |
| 17 | COL15A1 | collagen, type XV, alpha 1 | 3.81 |
| 18 | KITLG | KIT ligand | 3.80 |
| 19 | MCTP1 | multiple C2 domains, transmembrane 1 | 3.76 |
| 20 | SERPINB2 | serpin peptidase inhibitor, clade B (ovalbumin), member 2 | 3.74 |
| 21 | SNORD116-18 | small nucleolar RNA, C/D box 116-18 | 3.50 |
| 22 | OBFC2A | oligonucleotide/oligosaccharide-binding fold containing 2A | 3.35 |
| 23 | TM6SF1 | transmembrane 6 superfamily member 1 | 3.32 |
| 24 | HTN3 | histatin 3 | 3.31 |
| 25 | IL8 | interleukin 8 | 3.28 |
| 26 | MAL2 | mal, T-cell differentiation protein 2 | 3.26 |
| 27 | COMT | catechol-O-methyltransferase | 3.16 |
| 28 | PCDHB8 | protocadherin beta 8 | 3.15 |
| 29 | FBN2 | fibrillin 2 | 3.13 |
| 30 | SLC16A9 | solute carrier family 16, member 9 (monocarboxylic acid transporter 9) | 3.13 |
| 31 | GPR87 | G protein-coupled receptor 87 | 3.10 |
| 32 | GPR116 | G protein-coupled receptor 116 | 3.09 |
| 33 | CYFIP2 | cytoplasmic FMR1 interacting protein 2 | 3.08 |
| 34 | AK4 | adenylate kinase 4 | 3.06 |
| 35 | SCG5 | secretogranin V (7B2 protein) | 3.04 |
| 36 | MAP2 | microtubule-associated protein 2 | 3.01 |
